# Identification of a Novel lincRNA-p21-miR-181b-PTEN Signaling Cascade in Liver Fibrosis

**DOI:** 10.1155/2016/9856538

**Published:** 2016-08-16

**Authors:** Fujun Yu, Zhongqiu Lu, Bicheng Chen, Peihong Dong, Jianjian Zheng

**Affiliations:** ^1^Department of Infectious Diseases, The First Affiliated Hospital of Wenzhou Medical University, Wenzhou 325000, China; ^2^Department of Gastroenterology, Songjiang Hospital Affiliated Shanghai First People's Hospital, Shanghai Jiao Tong University, Shanghai 201600, China; ^3^Department of Gastroenterology, Shanghai Songjiang Hospital Affiliated to Nanjing Medical University, Nanjing 210029, China; ^4^Emergency Department, The First Affiliated Hospital of Wenzhou Medical University, Wenzhou 325000, China; ^5^Key Laboratory of Surgery, The First Affiliated Hospital of Wenzhou Medical University, Wenzhou 325000, China

## Abstract

Previously, we found that long intergenic noncoding RNA-p21 (lincRNA-p21) inhibits hepatic stellate cell (HSC) activation and liver fibrosis via p21. However, the underlying mechanism of the antifibrotic role of lincRNA-p21 in liver fibrosis remains largely unknown. Here, we found that lincRNA-p21 expression was significantly downregulated during liver fibrosis. In LX-2 cells, the reduction of lincRNA-p21 induced by TGF-*β*1 was in a dose- and time-dependent manner. lincRNA-p21 expression was reduced in liver tissues from patients with liver cirrhosis when compared with that of healthy controls. Notably, lincRNA-p21 overexpression contributed to the suppression of HSC activation. lincRNA-p21 suppressed HSC proliferation and induced a significant reduction in *α*-SMA and type I collagen. All these effects induced by lincRNA-p21 were blocked down by the loss of PTEN, suggesting that lincRNA-p21 suppressed HSC activation via PTEN. Further study demonstrated that microRNA-181b (miR-181b) was involved in the effects of lincRNA-p21 on HSC activation. The effects of lincRNA-p21 on PTEN expression and HSC activation were inhibited by miR-181b mimics. We demonstrated that lincRNA-p21 enhanced PTEN expression by competitively binding miR-181b. In conclusion, our results disclose a novel lincRNA-p21-miR-181b-PTEN signaling cascade in liver fibrosis and suggest lincRNA-p21 as a promising molecular target for antifibrosis therapy.

## 1. Introduction

Liver fibrosis, characterized by enhanced extracellular matrix (ECM), represents the common responses of the liver to various chronic injuries. It is well known that hepatic stellate cells (HSCs) are crucial mediators of liver fibrosis [1, 2]. In normal liver tissue, HSCs with abundant vitamin A stores are quiescent. Once activated, they will lose their vitamin A stores and become proliferative and fibrogenic. Currently, efficiently abrogating HSC activation is considered as a potential therapeutic strategy for treating liver fibrosis.

MicroRNAs (miRNAs) are highly conserved noncoding RNAs of ~22 nucleotides that regulate gene expression by binding to the 3′-untranslated region (3′-UTR) of the target gene mRNAs to repress protein translation or induce mRNA degradation [3]. Numerous studies have shown that miRNAs participate in the regulation of diverse biological processes including development and differentiation, immune response, metabolism, cell proliferation, and apoptosis [4]. Notably, miRNAs are involved in the pathogenesis and progression of liver fibrosis [5–7]. For instance, miR-29b overexpression upregulates phosphatase and tensin homologue deleted on chromosome 10 (PTEN) expression via inhibiting PTEN hypermethylation, leading to the suppression of HSC activation [7].

Long noncoding RNAs (lncRNAs) are transcribed RNA molecules (>200 nucleotides in length) that structurally resemble mRNAs but do not encode proteins [8]. Emerging data have reported that lncRNAs are involved in a wide range of biological processes, such as proliferation, apoptosis, and differentiation [9]. Recently, lncRNAs are reported to be involved in the progression of liver fibrosis [10, 11]. For example, we previously found that long intergenic noncoding RNA-p21 (lincRNA-p21) can act as an antifibrotic factor in liver fibrosis via p21 [11]. However, the underlying mechanism of the antifibrotic role of lincRNA-p21 in liver fibrosis remains largely unknown. Bian et al. found that DNA methyltransferase1- (DNMT1-) mediated PTEN hypermethylation contributes to HSC activation and liver fibrogenesis, suggesting that PTEN is a negative regulator of liver fibrosis [12]. However, the roles of PTEN in the effects of lincRNA-p21 on HSC activation had never been studied. In this study, we aimed to investigate whether PTEN plays a crucial role in the effects of lincRNA-p21 on liver fibrosis.

## 2. Materials and Methods 

### 2.1. Materials

Transforming growth factor-*β*1 (TGF-*β*1) was purchased from R&D Systems (Shanghai, China). Adenoviral vectors expressing a control scrambled sequence (Ad-Ctrl) and adenoviral vectors expressing lincRNA-p21 (Ad-lincRNA-p21) were purchased from GenePharma biotechnology (Shanghai, China). Antibodies against type I collagen, *α*-smooth muscle actin (*α*-SMA), PTEN, and GAPDH were obtained from Abcam (Cambridge, MA, USA). Chemically synthesized RNAs including negative control (miR-NC), miR-181b mimics, and miR-181b inhibitor were obtained from GenePharma biotechnology. For transfection, cells were transfected with 1 *μ*g of the chemically synthesized RNA using Lipofectamine 2000 (Invitrogen, USA).

### 2.2. Human Specimens

Written informed consent was received from all patients prior to liver tissues. In this study, 15 healthy controls and 15 liver cirrhosis patients undergoing partial liver resection or liver biopsy were selected from the First Affiliated Hospital of Wenzhou Medical University. Liver cirrhosis was diagnosed by liver biopsy and/or a typical appearance of the liver on abdominal ultrasound and/or computed tomography scan. This study was performed in compliance with the Declaration of Helsinki and approved by the Ethics Committee of the First Affiliated Hospital of Wenzhou Medical University.

### 2.3. Cell Culture

Human LX-2 cell strain was obtained from JENNIO Biological Technology (Guangdong, China). It was cultured in DMEM containing 10% fetal bovine serum, 100 U/mL penicillin G sodium salt, and 100 U/mL streptomycin sulfate (Gibco, Carlsbad, CA). The cells were grown in a 37°C incubator with 5% CO_2_. Exponentially growing cells were seeded in a six-well plate at a density of 1 × 10^6^ cells/well; then the cells were transduced with the Ad-lincARNA-p21 or Ad-Ctrl for 48 h. Cells were also treated with TGF-*β*1 for different experiment purposes. Cells were harvested for RNA/miRNA isolation, and whole cell extracts were subjected to western blot analysis.

### 2.4. RNA Interference Analysis

RNA interference experiments were performed using Lipofectamine 2000 (Invitrogen) in accordance with the manufacturer's instructions. PTEN siRNA1 (sense 5′-UCUCAAACUUCCAUCAUGGCU-3′; antisense 5′-CCAUGAUGGAAGUUUGAGAGU-3′), PTEN siRNA2 (sense 5′-UGAUAUCUCCUUUUGUUUCUG-3′; antisense 5′-GAAACAAAAGGAGAUAUCAAG-3′), and scrambled siRNA (negative control) were designed and synthesized by GenePharma biotechnology. siRNAs were transfected into cells at a final concentration of 100 nM.

### 2.5. Quantitative Real-Time PCR

Total RNA was extracted from human liver tissues and LX-2 cells using miRNeasy Mini kit (Qiagen, Valencia, CA, USA) according to manufacturer's instructions. Fifty nanograms of total RNA was reverse-transcribed to cDNA using the ReverTra Ace qPCR RT Kit (Toyobo, Osaka, Japan). Gene expression was measured by real-time PCR using cDNA, SYBR Green real-time PCR Master Mix (Toyobo, Osaka, Japan). The primers of lincRNA-p21, alpha-1 (I) collagen (Col1A1), *α*-SMA, GAPDH, and U6 were designed as described previously [13, 14]. The primers used for PTEN were 5′-CAGGATACGCGCTCGGC-3′ and 5′-ACAGCGGCTCAACTCTCAAA-3′. To detect the expressions of miR-21, miR-24, miR-32, miR-93, miR-153, miR-181b, miR-205, and miR-214, the RT reaction was performed using the TaqMan MicroRNA Assay (Applied Biosystems, Foster City, CA) according to the manufacturer's instructions. The GAPDH (Applied Biosystems, Foster City, CA) level was used to normalize the relative abundance of lincRNA-p21 and mRNAs. U6 snRNA (Applied Biosystems, Foster City, CA) was used to normalize the relative abundance of miRNAs. The expression levels (2^−ΔΔCt^) of lincRNA-p21, mRNAs, and miRNAs were calculated as described previously [15].

### 2.6. Protein Extraction and Western Blot Assay

The protein concentration of samples was determined by a BCA protein assay kit (Beyotime Biotechnology, Jiangsu, China). Total proteins (30–50 *μ*g) were separated by SDS-PAGE and blotted onto a PVDF membrane (Millipore Corp, Billerica, MA, USA). After blocking, nitrocellulose blots were incubated for 1 h with primary antibodies diluted in TBS/Tween20 (0.075%) containing 3% Marvel. Rabbit polyclonal antibodies against type I collagen and PTEN were diluted 1 : 1000, and mouse monoclonal antibodies against *α*-SMA and GAPDH were used at 1 : 2000. The membranes were washed 3 times with TBS/Tween20 (0.075%) containing 3% Marvel, followed by incubation with HRP-conjugated secondary antibodies (1 : 5000) at 37°C for 1 h. The antigen-antibody complex was developed by enhanced chemiluminescence, exposed in the dark room and analyzed for integral absorbance (IA) of the protein bands using quantitative software, Quantity One 4.4.

### 2.7. Cell Proliferation Assay

Cells were seeded in 96-well plates at a density of 1 × 10^3^ cells per well and cultured for 24 h. Cell were transduced with Ad-lincRNA-p21 for 48 h and treated with PTEN siRNA or miR-181b mimics for additional 48 h. Then, cell proliferation was assessed using CCK-8 (Dojindo, Kumamoto, Japan) according to manufacturer's instructions. Absorbance values for each well were determined at 450 nm using a microplate reader (Molecular Devices, Sunnyvale, CA, USA). All experiments were performed in triplicate and repeated at least three times.

### 2.8. Luciferase Activity Assay

According to RNA22 analysis, oligonucleotides containing human lincRNA-p21 3′UTR target sequence were annealed and cloned into the pmirGLO plasmids (Promega, Madison, WI, USA) to generate the pmioGLO-lincRNA-p21 vector: lincRNA-p21-3′UTR for miR-181b (position of 1296–1318) forward, 5′-CCCTCCGACAGGAGTCTCA-3′, and reverse, 5′-TTGAGAGAGATGCACAGCCAG-3′. Empty plasmid pmirGLO was regarded as a negative control. Luciferase reporter plasmids plus miR-181b mimics or miR-NC were cotransfected into LX-2 cells using Lipofectamine 2000 (Invitrogen). Forty-eight hours after transfection, relative luciferase activity was examined in a luminometer using a Dual-Luciferase Reporter Assay System (Promega).

### 2.9. Statistical Analysis

Data from at least three independent experiments were expressed as the mean ± SD. Comparisons between two groups and multiple groups were made using Student's* t*-test and one-way analysis of variance, respectively. Correlation between the expressions of miR-181b and lincRNA-p21 in liver tissues was examined by Pearson's correlation coefficient. *P* < 0.05 was considered significant. All statistical analyses were performed with SPSS software (version 13; SPSS, Chicago, IL).

## 3. Results

### 3.1. lincRNA-p21 Is Downregulated in TGF-*β*1-Treated HSCs and Human Liver Cirrhosis

**Figure 1:**
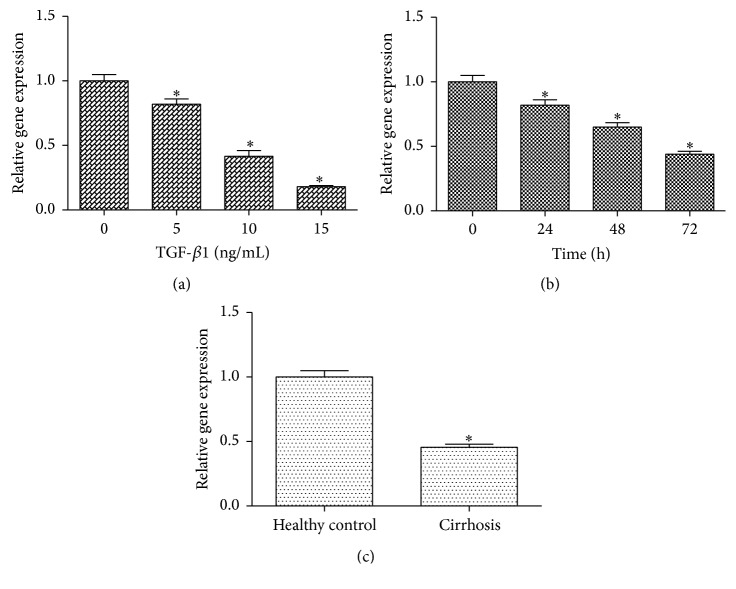
lincRNA-p21 is downregulated in TGF-*β*1-treated HSCs and human liver fibrosis. (a) The downregulation of lincRNA-p21 expression was dose-dependently induced by TGF-*β*1. LX-2 cells were treated with TGF-*β*1 (0, 5, 10, and 15 ng/mL) for 24 h. (b) The downregulation of lincRNA-p21 expression was time-dependently induced by TGF-*β*1. LX-2 cells were treated with TGF-*β*1 (5 ng/mL) for 0, 24, 48, and 72 h. (c) Expression of lincRNA-p21 in liver tissues of healthy controls (*n* = 15) and cirrhotic patients (*n* = 15). Each value is the mean ± SD of three experiments. ^*∗*^ *P* < 0.05 compared with the control.

In the initiation and progression of liver fibrosis, HSC activation is considered as a vital event [16]. HSC activation can be triggered by various inflammatory cytokines, of which TGF-*β*1 is recognized as the main profibrogenic mediator [17]. In our experiments, immortalized human stellate cells, LX-2, were treated with different concentrations of TGF-*β*1, and the expression of lincRNA-p21 was detected. There was a significant reduction in lincRNA-p21 level as the increased concentrations of TGF-*β*1 (from 0 to 15 ng/mL), suggesting that lincRNA-p21 was reduced by TGF-*β*1 in a dose-dependent manner (Figure 1(a)). Next, lincRNA-p21 expression was detected at 0, 24, 48, and 72 h in TGF-*β*1-treated HSCs. Expression was significantly decreased with time, with the lowest level observed at 72 h (Figure 1(b)), clearly indicating lincRNA-p21 expression was reduced by TGF-*β*1 in a time-dependent manner. lincRNA-p21 expression was additionally detected in human normal liver tissues and cirrhotic tissues. Compared with the control, lincRNA-p21 expression was reduced in the cirrhotic samples (Figure 1(c)). These data collectively suggest that lincRNA-p21 is downregulated during liver fibrosis.

### 3.2. The Effects of lincRNA-p21 Overexpression on HSC Activation

**Figure 2:**
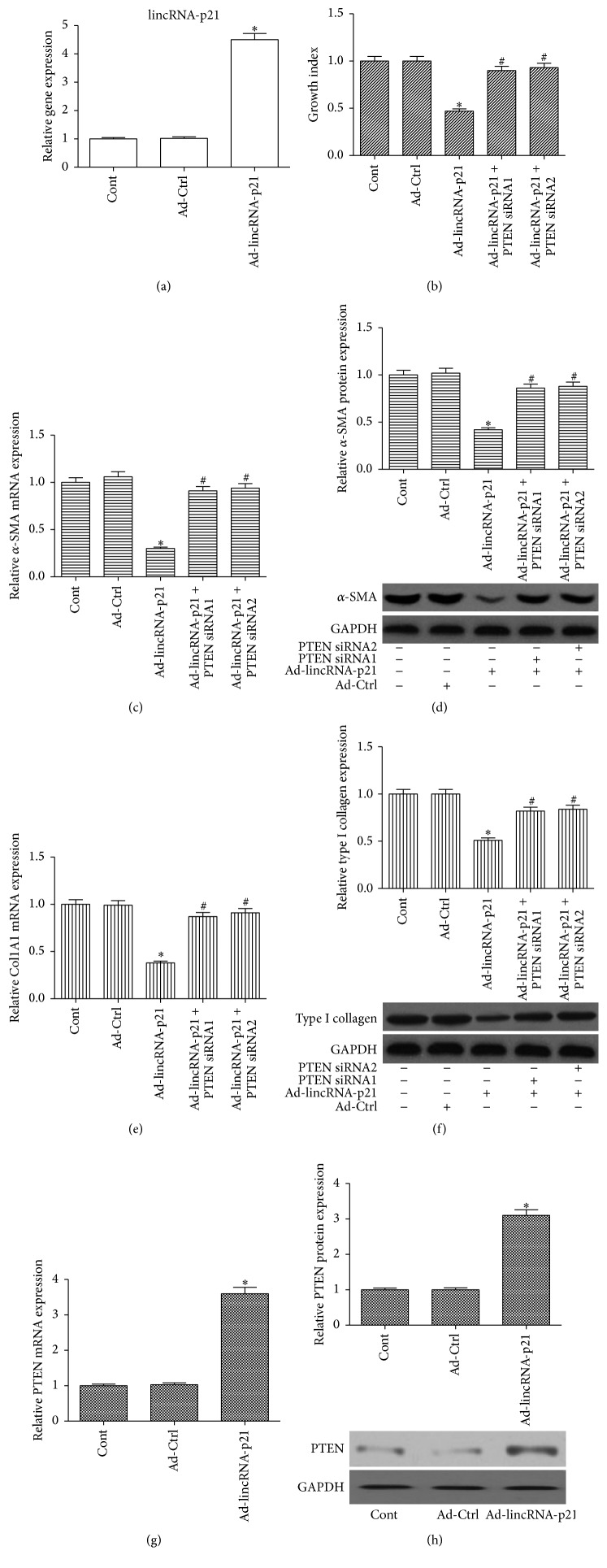
Effects of lincRNA-p21 overexpression on cell proliferation, *α*-SMA, type I collagen, and PTEN in LX-2 cells. Cells were transduced with Ad-lincRNA-p21 for 48 h and treated with PTEN siRNA for additional 48 h. (a) lincRNA-p21 was detected in cells transduced with Ad-lincRNA-p21. Overexpression of lincRNA-p21 suppressed cell proliferation (b), *α*-SMA mRNA (c), *α*-SMA protein (d), Col1A1 mRNA (e), and type I collagen (f), which were almost blocked down by PTEN siRNA. Cell proliferation was assessed by CCK-8 assay. PTEN mRNA (g) and protein (h) expressions were upregulated by Ad-lincRNA-p21. GAPDH was used as internal control. Each value is the mean ± SD of three experiments. ^*∗*^ *P* < 0.05 compared with the control and ^#^ *P* < 0.05 compared with Ad-lincRNA-p21 group.

HSC activation is characterized by enhanced cell proliferation, overproduction of ECM, and* de novo* synthesis of *α*-SMA [18]. To investigate whether lincRNA-p21 overexpression inhibits HSC activation, LX-2 cells were transduced with Ad-lincRNA-p21 to increase lincRNA-p21 level. Compared with the control, the delivery of Ad-lincRNA-p21 effectively induced the elevation of lincRNA-p21 level (Figure 2(a)). Next, the effects of lincRNA-p21 on cell proliferation were examined in HSCs transduced with Ad-lincRNA-p21. As indicated by the results of CCK-8 assay, lincRNA-p21 overexpression resulted in a significant reduction in HSC proliferation (Figure 2(b)). To confirm the effects of lincRNA-p21 overexpression on HSC transdifferentiation, the mRNA and protein levels of *α*-SMA in LX-2 cells were detected by real-time PCR and immunoblot analysis, respectively. The results of real-time PCR showed that overexpression of lincRNA-p21 induced a significant reduction in *α*-SMA mRNA expression (Figure 2(c)). Consistent with the mRNA data, immunoblot assays revealed that lincRNA-p21 inhibited *α*-SMA protein expression (Figure 2(d)). Then, the effects of lincRNA-p21 on collagen expression were examined. Ad-lincRNA-p21 caused a significant reduction in Col1A1 mRNA expression (Figure 2(e)). In addition, immunoblot assays demonstrated that type I collagen was suppressed by Ad-lincRNA-p21 (Figure 2(f)). These data suggest that HSC activation can be suppressed by lincRNA-p21.

### 3.3. lincRNA-p21 Inhibits HSC Activation via PTEN

PTEN has been reported to function as a “fibrotic suppressor” gene in liver fibrosis [12]. To investigate whether PTEN was involved in the effects of lincRNA-p21 on HSC activation, PTEN expression was detected in HSCs transduced with Ad-lincRNA-p21. Notably, the mRNA and protein expressions of PTEN were enhanced by overexpression of lincRNA-p21 (Figures 2(g) and 2(h)), indicating that PTEN may play a role in the effects of lincRNA-p21 on liver fibrosis. To further confirm whether PTEN was responsible for the antifibrotic effects induced by lincRNA-p21, lincRNA-p21-overexpressing cells were treated with PTEN siRNA. The suppressive effect of PTEN siRNA on PTEN level was examined. As shown in Figure S1A (in Supplementary Material available online at http://dx.doi.org/10.1155/2016/9856538), both PTEN siRNA1 and PTEN siRNA2 caused a significant reduction in PTEN protein level. Then, the expressions of *α*-SMA and collagen were examined in lincRNA-p21-overexpressing cells with PTEN siRNA. It was found that reduced HSC proliferation caused by lincRNA-p21 was blocked down by PTEN siRNA (Figure 2(b)). In addition, the reduced *α*-SMA and type I collagen caused by lincRNA-p21 were restored by the silencing of PTEN (Figures 2(c)–2(f)). These results suggest that HSC activation is suppressed by lincRNA-p21, at least in part, via PTEN.

### 3.4. miR-181b Is Involved in the Effects of lincRNA-p21 on HSC Activation

**Figure 3:**
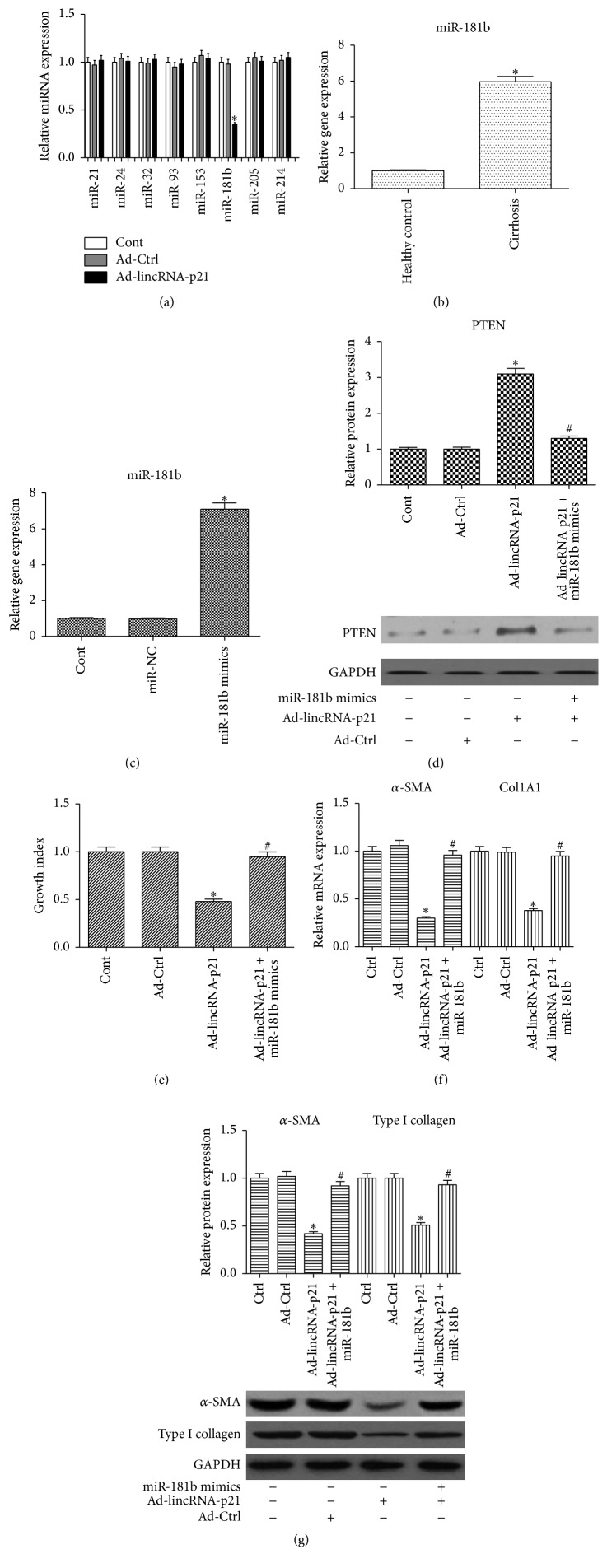
miR-181b is involved in the effects of lincRNA-p21 on PTEN expression and HSC activation. Cells were transduced with Ad-lincRNA-p21 for 48 h and treated with miR-181b mimics for additional 48 h. (a) Expressions of miR-21, miR-24, miR-32, miR-93, miR-153, miR-181b, miR-205, and miR-214 were detected in cells transduced with lincRNA-p21. (b) Expression of miR-181b in liver tissues of healthy controls (*n* = 15) and cirrhotic patients (*n* = 15). (c) Expression of miR-181b in miR-181b mimics group. (d) lincRNA-p21-induced PTEN was inhibited by miR-181b. (e) The effect of Ad-lincRNA-p21 on cell proliferation was suppressed by miR-181b. (f) The reduced mRNA expressions of *α*-SMA and Col1A1 by Ad-lincRNA-p21 were inhibited by miR-181b. (g) The reduced protein expressions of *α*-SMA and type I collagen by Ad-lincRNA-p21 were inhibited by miR-181b. GAPDH was used as internal control. Each value is the mean ± SD of three experiments. ^*∗*^ *P* < 0.05 compared with the control and ^#^ *P* < 0.05 compared with Ad-lincRNA-p21 group.

Using bioinformatic analysis (TargetScan), PTEN is predicted as a target of miRNAs. In theory, PTEN can be regulated by them. Many studies have demonstrated that miRNAs including miR-21, miR-24, miR-32, miR-93, miR-153, miR-205, and miR-214 can regulate PTEN expression as a target in human diseases [19–25]. Our previous study found that miR-181b promotes HSC activation via PTEN/Akt pathway [26]. Recently, lncRNAs have been reported to act as competing endogenous RNAs (ceRNAs) to sponge miRNAs, consequently modulating the derepression of miRNA targets [9]. We hypothesized that lincRNA-p21-mediated PTEN expression may be through a ceRNA mechanism. Next, these miRNAs were detected in lincRNA-p21-overexpressing cells. It was found that miR-181b level was reduced by lincRNA-p21, whereas others were not (Figure 3(a)). Compared with the healthy controls, miR-181b level was enhanced in the cirrhotic samples (Figure 3(b)), indicating that miR-181b may be inversely correlated with lincRNA-p21 expression in liver fibrosis. To confirm whether miR-181b was involved in the effects of lincRNA-p21 on PTEN level and HSC activation, lincRNA-p21-overexpressing cells were transfected with miR-181b mimics. Compared with the control, there was a significant increase in miR-181b level in miR-181b mimics group (Figure 3(c)). Interestingly, increased PTEN protein level induced by lincRNA-p21 was inhibited by miR-181b mimics (Figure 3(d)). By contrast, as shown in Fig.S1B, lincRNA-p21-induced PTEN protein level was further enhanced by miR-181b inhibitor. Moreover, lincRNA-p21 overexpression resulted in a significant reduction in HSC proliferation, *α*-SMA, and type I collagen expressions, which were all blocked down by miR-181b mimics (Figures 3(e)–3(g)). All the data suggest that miR-181b is involved in the antifibrotic effects of lincRNA-p21.

### 3.5. lincRNA-p21 Is a Target of miR-181b

**Figure 4:**
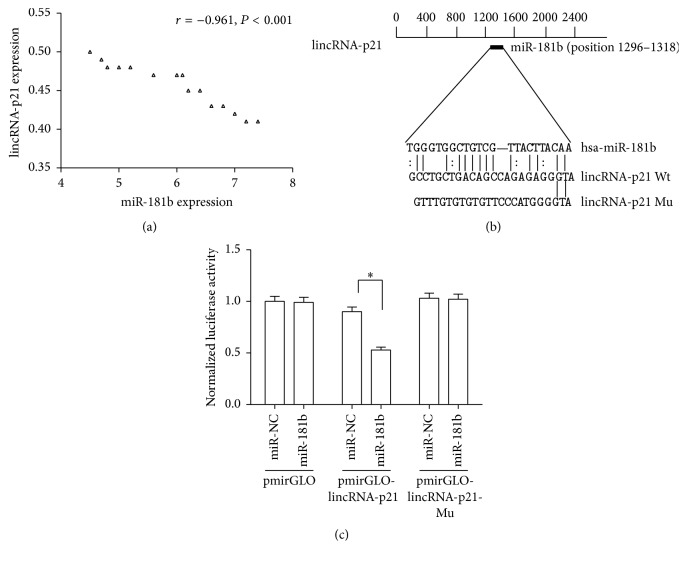
The effect of lincRNA-p21 on PTEN expression is through competitively binding miR-181b. (a) Correlation between lincRNA-p21 level and miR-181b expression in liver tissue samples from cirrhotic patients (*n* = 15) was subjected to Pearson correlation analysis. (b) Schematic diagram of the miR-181b binding site in the lincRNA-p21 based on RNA22 software. (c) Relative luciferase activities of luciferase reporters bearing wild-type or mutant lincRNA-p21 were analyzed 48 h following transfection with the indicated miR-181b mimics or miR-NC in LX-2 cells. Each value is the mean ± SD of three experiments. ^*∗*^ *P* < 0.05.

In the liver tissue samples from cirrhotic patients, there was a strong negative correlation between lincRNA-p21 level and miR-181b expression (*r* = −0.961, *P* < 0.001) (Figure 4(a)). There might be a relation between lincRNA-p21 and miR-181b, and this hypothesis was confirmed by luciferase reporter assays. Using bioinformatic analysis (RNA22), it was found that lincRNA-p21 contains one target site for miR-181b (Figure 4(b)). Using pmirGLO construct, we generated a lincRNA-p21 luciferase reporter containing the miR-181b-binding sites (pmirGLO-lincRNA-p21) or mutated sites (pmirGLO-lincRNA-p21-Mu) (Figure 4(b)). miR-181b mimics induced a reduction in luciferase activity of pmirGLO-lincRNA-p21 without affecting that of pmirGLO-lincRNA-p21-Mu (Figure 4(c)). lincRNA-p21 is confirmed as a target of miR-181b. All the data suggest that lincRNA-p21 enhances PTEN expression through competitively binding miR-181b.

## 4. Discussion

The results of the present study showed that PTEN expression was enhanced by lincRNA-p21 via the regulation of miR-181b. HSC activation was significantly suppressed by overexpression of lincRNA-p21, including the reduction in HSC proliferation, ECM protein, and *α*-SMA expression. Without the enhanced PTEN caused by lincRNA-p21, all these effects were blocked down, suggesting that lincRNA-p21 suppresses liver fibrosis, at least in part, via PTEN. Notably, further studies showed that enhanced PTEN protein level and the antifibrotic effects induced by lincRNA-p21 were blocked down by miR-181b. As confirmed by luciferase activity assays, lincRNA-p21 was confirmed as a target of miR-181b. Combined with the data above, we demonstrated that lincRNA-p21 contributes to the suppression of liver fibrosis via miR-181b-mediated PTEN.

LncRNAs, lacking significant protein-coding capacity, can regulate a wide range of biological processes through diverse molecular mechanisms including chromatin modification, transcriptional regulation, and posttranscriptional regulation [9, 27, 28]. Moreover, lncRNAs can regulate miRNAs-mediated cellular processes by sponging miRNAs via a ceRNA mechanism. For instance, metastasis-associated lung adenocarcinoma transcript 1 (MALAT1) contributes to liver fibrosis via the regulation of RAS-related C3 botulinum substrate 1 (Rac1) and miR-101b [29]. In the past few years, the roles and functions of lncRNAs in human diseases have been widely explored. For example, the G_1_/S checkpoint is reported to be regulated by lincRNA-p21 via p21* in cis* [30]. miRNA-regulated delivery of lincRNA-p21 suppresses *β*-catenin signaling and tumorigenicity of colorectal cancer stem cells [31]. Our previous study showed that lincRNA-p21 inhibits HSC activation and liver fibrosis via p21 [11]. In our previous study, the roles of lincRNA-p21 in liver fibrosis were explored in mouse primary HSCs and mice. But the present study focused on the roles of lincRNA-p21 in immortalized human stellate cells, LX-2. Interestingly, a novel antifibrosis signaling pathway was identified in the effects of lincRNA-p21 in liver fibrosis. Our data demonstrated that lincRNA-p21 inhibits liver fibrosis through sponging miR-181b in LX-2 cells.

PTEN, as a tumor suppressor, is often deregulated in various cancers. Both phosphatidylinositol 3-kinase (PI3K)/Akt and extracellular signal-regulated kinase (ERK) pathways can be regulated by PTEN through its lipid phosphatase and protein tyrosine phosphatase activity, respectively [32, 33]. The loss of PTEN results in the activation of PI3K/Akt and ERK pathways, leading to a reduction in cellular apoptosis and an increase in mitogen signaling [34, 35]. Recent study shows that PTEN is downregulated during liver fibrosis and considered as a negative regulator of liver fibrosis [12]. Overexpression of PTEN leads to the suppression of HSC activation and liver fibrosis [36]. Recently, miR-181b has been reported to contribute to the activation of HSCs through its targets such as p27 [37]. Therefore, miR-181b may play a profibrotic role in liver fibrosis. Our previous study demonstrated that PTEN is a target of miR-181b and HSCs can be activated by miR-181b via PTEN/Akt pathway [26]. Based on our previous study and the role of PTEN in liver fibrosis, whether miR-181b-mediated PTEN was involved in the effects of lincRNA-p21 in liver fibrosis was further explored. At the present study, our results showed that lincRNA-p21 inhibits HSC activation, at least in part, via miR-181b-mediated PTEN. Our data identify a novel lincRNA-p21/miR-181b/PTEN signaling cascade in liver fibrosis. However, the functions of lincRNA-p21 in HSC may be more complex than what we imagine, and further functional analysis should be performed to determine the precise role of lincRNA-p21 in HSC.

In conclusion, our results provide new insights that lincRNA-p21 inhibits the progression of liver fibrosis via PTEN. Moreover, our data disclose a novel lincRNA-p21/miR-181b/PTEN signaling cascade in liver fibrosis. The downregulation of lincRNA-p21 in patients with cirrhosis suggests that it may be potential diagnostic biomarkers for cirrhosis.

## Supplementary Material

Fig.S1 The effects of PTEN siRNA, Ad-lincRNA-p21 or miR-181b inhibitor on the protein expression of PTEN. (A) PTEN protein was reduced by PTEN siRNA1 or siRNA2. Cells were transfected with PTEN siRNA for 48 h. (B) PTEN protein was increased by Ad-lincRNA-p21, which was further enhanced by miR-181b inhibitor. Cells were transduced with Ad-lincRNA-p21 for 48 h and treated with miR-181b inhibitor for additional 48 h. GAPDH was used as internal control. Each value is the mean ± SD of three experiments. ∗P<0.05 compared with the control and #P<0.05 compared with Ad-lincRNA-p21 group.
