# Supplementary figures and images for: Identification of a Novel lincRNA-p21-miR-181b-PTEN Signaling Cascade in Liver Fibrosis
Source: Mediators Inflamm. 2016 Aug 16;2016:9856538. doi: 10.1155/2016/9856538 (PMC5004029; doi:10.1155/2016/9856538)

A

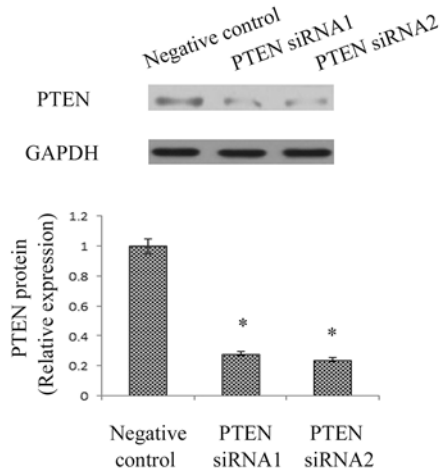

B

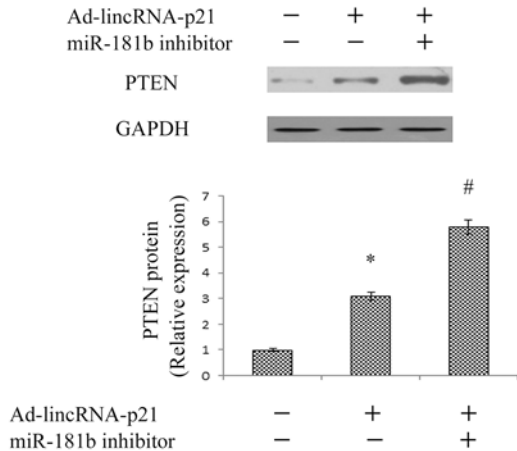

Supplement: Supplementary file 1 — Fig.S1 The effects of PTEN siRNA, Ad-lincRNA-p21 or miR-181b inhibitor on the protein expression of PTEN. (A) PTEN protein was reduced by PTEN siRNA1 or siRNA2. Cells were transfected with PTEN siRNA for 48 h. (B) PTEN protein was increased by Ad-lincRNA-p21, which was further enhanced by miR-181b inhibitor. Cells were transduced with Ad-lincRNA-p21 for 48 h and treated with miR-181b inhibitor for additional 48 h. GAPDH was used as internal control. Each value is the mean ± SD of three experiments. ∗P<0.05 compared with the control and #P<0.05 compared with Ad-lincRNA-p21 group. [file 9856538.f1.pdf]
